# Measuring Brain Complexity During Neural Motor Resonance

**DOI:** 10.3389/fnins.2018.00758

**Published:** 2018-10-30

**Authors:** Brandon M. Hager, Albert C. Yang, Jennifer N. Gutsell

**Affiliations:** ^1^Department of Psychology, Brandeis University, Waltham, MA, United States; ^2^Division of Interdisciplinary Medicine and Biotechnology, Beth Israel Deaconess Medical Center, Harvard Medical School, Boston, MA, United States; ^3^Department of Psychology, Neuroscience Program, and Volen National Center for Complex Systems, Brandeis University, Waltham, MA, United States

**Keywords:** motor resonance, complexity, network connectivity, EEG, mu suppression

## Abstract

**Background:** EEG mu-desynchronization is an index of motor resonance (MR) and is used to study social interaction deficiencies, but finding differences in mu-desynchronization does not reveal how nonlinear brain dynamics are affected during MR. The current study explores how nonlinear brain dynamics change during MR. We hypothesized that the complexity of the mu frequency band (8–13 Hz) changes during MR, and that this change would be frequency specific. Additionally, we sought to determine whether complexity at baseline and changes in complexity during action observation would predict MR and changes in network dynamics.

**Methods:** EEG was recorded from healthy participants (*n* = 45) during rest and during an action observation task. Baseline brain activity was measured followed by participants observing videos of hands squeezing stress balls. We used multiscale entropy (MSE) to quantify the complexity of the mu rhythm during MR. We then performed *post-hoc* graph theory analysis to explore whether nonlinear dynamics during MR affect brain network topology.

**Results:** We found significant mu-desynchronization during the action observation task and that mu entropy was significantly increased during the task compared to rest, while gamma, beta, theta, and delta bands showed decreased entropy. Moreover, resting-state entropy was significantly predictive of the degree of mu desynchronization. We also observed a decrease in the clustering coefficient in the mu band only and a significant decrease in global alpha efficiency during action observation. MSE during action observation was strongly correlated with alpha network efficiency.

**Conclusions:** The current findings suggest that the desynchronization of the mu wave during MR results in a local increase of mu entropy in sensorimotor areas, potentially reflecting a release from alpha inhibition. This release from inhibition may be mediated by the baseline MSE in the mu band. The dynamical complexity and network analysis of EEG may provide a useful addition for future studies of MR by incorporating measures of nonlinearity.

## Introduction

There has been great interest in the idea of neural mirroring–neural simulation of the actions and experiences of others while observing them–as a central process contributing to action understanding and experience sharing. Using electroencephalogram (EEG) to record the desynchronization of the mu wave (8–13 Hz) over sensorimotor regions is gaining popularity as a measure of neural simulation (e.g., Cheng et al., 2008; Pineda and Hecht, 2009; Gutsell and Inzlicht, 2010; Perry et al., 2010; Fabi and Leuthold, 2017; Li et al., 2017), including in the clinical setting (Oberman et al., 2008, 2013; Fan et al., 2010; Mitra et al., 2014; Minichino et al., 2016). Several researchers have used mu desynchronization to investigate impaired social and emotional processing in disorders like schizophrenia and autism (Oberman et al., 2005; McCormick et al., 2012; Horan et al., 2014; Brown et al., 2016). However, studies using mu desynchronization during action observation, have relied solely on measuring attenuation of the mu power spectrum. Utilizing the Fast Fourier transform (FFT) to obtain the average power of pre-selected frequency components or the Morlet wavelet transform to obtain frequency information at a specific moment in time, can only provide information on power spectrum changes and fails to address potentially important changes to nonlinear brain dynamics. The current study measured changes to neural complexity and network connectivity that occur during action observation, and sought to assess how these changes are related to, and might supplement, the linear changes observed in the mu power spectrum.

Viewing the actions of another person triggers neural representations similar to when actually performing the same action (di Pellegrino et al., 1992; Rizzolatti and Craighero, 2004; Rizzolatti, 2005), and such motor resonance (MR) is thought to transform visual information about the action including basic intentions into knowledge (Rizzolatti et al., 2009; Liu et al., 2016). EEG mu desynchronization is considered a valid measure of MR (see Fox et al., 2015 for a meta-analysis and Hobson and Bishop, 2016, for a critical perspective). The mu-rhythm can be picked up over the sensorimotor cortex at central electrodes (C3, C1, CZ, C2, and C4 electrodes), and its desynchronization is correlated with activation in areas thought to be part of the human mirror system, including the dorsal premotor cortex, the primary somatosensory cortex, and the inferior parietal lobe (Perry and Bentin, 2009; Arnstein et al., 2011; Yin et al., 2016). Since the decrease in mu amplitude during mu desynchronization occurs because the number of synchronously active neurons firing at a frequency of 8–13 Hz decreases during action and action observation (Lopes da Silva, 1991), the underlying nonlinear patterns of activity should become more complex (non-randomly varying), and may provide information on the adaptability of a system or network of connections to a stimulus (McIntosh et al., 2008; Manor et al., 2010; Vakorin et al., 2011). Such nonlinear patterns contain information that is not accessible by spectral measures (Meyer-Lindenberg et al., 1998; Abásolo et al., 2006; Park et al., 2007; Mizuno et al., 2010), and the current study is a novel investigation of EEG signal during action observation.

A promising approach to analyzing nonlinear dynamics in the brain is multi scale entropy (MSE), which measures entropy over multiple time scales inherent in a time series (Costa et al., 2002). Sample entropy of each coarse-grained time series serves as an index of signal complexity by evaluating the occurrence of repetitive patterns. Thus, using MSE analysis in this study allows us to extract meaningful information about the changes to nonlinear dynamics that occur in the EEG signal during action observation. Moreover, changes in entropy indexed using MSE, are likely to indicate, to some degree, a change in the neural underpinnings of connectivity (Friston et al., 1995; Sporns et al., 2000; Takahashi et al., 2010). To validate this assumption, we also measured changes in connectivity across the scalp using graph theoretical analysis and tested their link to changes in entropy. More specifically, we used global efficiency and the cluster coefficient (graph theoretical measures), to provide information on local and global changes to functional connectivity that result from local changes in nonlinear dynamics.

In sum, the first goal of the current study was to capture changes in the complexity of the signal measured over the sensorimotor area during MR using MSE analysis. The second goal was to determine how resting state MSE relates to mu desynchronization during MR. Resting-state complexity has been previously used as a predictor for risk of developing ASD (Bosl et al., 2011), as well as predicting cognitive function in Alzheimer's disease (Mizuno et al., 2010). The finding by Mizuno et al. (2010), suggests that complexity at rest can predict one's adaptability to a future “engaged” or active state. Along the same lines, we expected the level of resting MSE measured in the mu frequency band, to be predictive of the amount of decrease, if any, in the mu power spectrum during action observation. The final goal of the study was to assess whether MR changes network communication in the gamma, beta, alpha, theta, and delta bands, and its relationship to the MSE measured over the sensorimotor area.

## The current research

We measured MR during action observation, using mu desynchronization, as part of a larger study that tested how perceptions of warmth and competence of target individuals affect MR. Participants first viewed personality ratings of varying combinations of the warmth and competence of a target person followed by a short video of ostensibly that person performing an action. We collapsed the action observation data across all warmth and competence conditions to assess mu desynchronization, MSE change, and network changes. We hypothesized that the level of MSE (average entropy across all scale factors) measured in the mu frequency band would increase during action observation relative to baseline, and that an increase would be linked to mu desynchronization, such that stronger desynchronization would be associated with a larger increase in MSE. Additionally, we tested whether changes to MSE during action observation was frequency-band specific by analyzing change in the MSE values in four other frequency bands (gamma, beta, theta, and delta). We hypothesized that the level of baseline MSE would predict the degree of mu desynchronization, thereby being a determinant of adaptability during action observation. Finally, we used graph theory analysis to assess the effect of mu desynchronization on network communication by analyzing global efficiency in the five mentioned frequency bands, and the cluster coefficient over the sensorimotor area, respectively.

## Methods

### Participants

Participants (*n* = 66) right-handed undergraduate college students had normal or corrected vision, and the ability to read and write fluently in English. Participants were all right-handed to ensure analogous cortical responses to videos of right hands performing simple motor activities.

All participants provided informed consent and participated in the research for course credit or financial compensation. Participants were excluded due to equipment failure resulting in too many non-functioning electrodes (cutoff of 5 non-functioning electrodes; *N* = 4), excessive EEG artifacts in the C3 electrode (*N* = 12; such as blinks, electromyogram (EMG), line noise, and visible signal drift), and visually excessive frontal alpha activity (*N* = 4) which suggests a participant might be sleeping, leading to a final sample of 45 participants (mean age = 18.814, SD = 0.958; 33 female).

### Procedure

The experimental protocol and data acquisition procedure were approved by the Brandeis Institutional Review Board. After EEG setup was complete, participants were asked to repeatedly squeeze a stress ball using their right hand for 15 s while we filmed their hand. This both created a baseline for brain activity during actual hand movement and reinforced the cover story that participants would be watching videos previously recorded from other participants. Participants then underwent a measure of baseline neural activity for which they sat completely still, first with their eyes closed for 1.5 min, then open for 1.5 min, then while watching a video of white noise for 1 min. The baseline recorded with eyes open was used to calculate the complexity of the mu rhythm at rest.

Following the baseline recording, participants were told that they would be taking a personality test. The test required them to rate themselves on a list of adjectives related to warmth and competence. They were then told that nine other people had previously taken the same test and had their hand movement recorded. EEG was recorded while participants viewed the action videos following the warmth and competence information. Finally, participants filled out a demographic questionnaire, were debriefed, thanked, and dismissed.

### Action observation task

**Figure 1:**
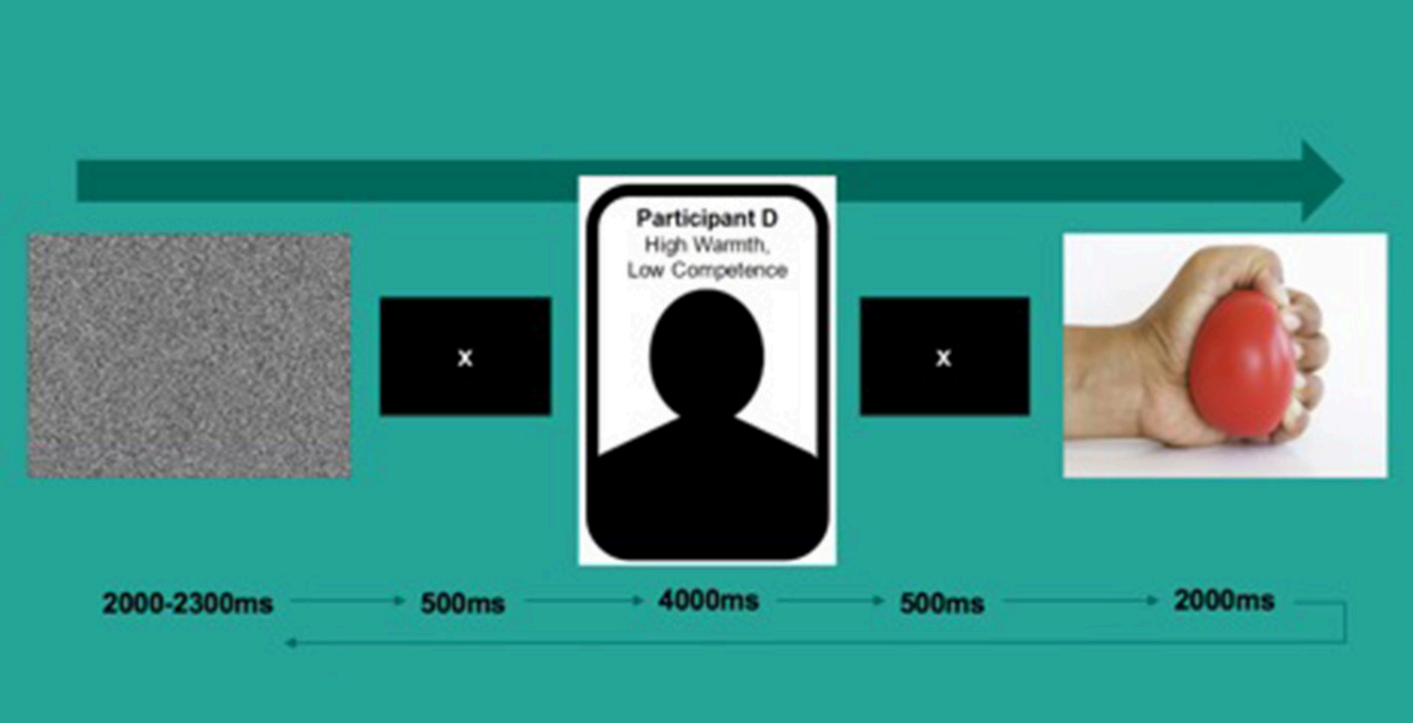
Depiction of a typical task trial.

The task consisted of 135 trials. During each trial, participants were first presented with a video of white noise for 2,000–2,300 ms followed by a fixation cross for 500 ms. They then viewed a screen with a target's name (e.g., “Participant A”), a simple silhouette and warmth/competence scores (e.g., “Warmth: High, Competence: Low”) for 4,000 ms, followed by a video of what was ostensibly that person's hand squeezing a yellow stress ball for 2,000 ms at the rate of approximately one squeeze per second (see Figure 1 for a depiction of a typical trial). Each hand was always paired with the same identity; all hands were White and with an even gender split (five female). The task was split into two blocks of ~15 min in length divided by a short break of a duration determined by the participant.

### EEG recording and processing

EEG was recorded from 32 active electrodes embedded in a stretch-lycra cap (ActiCap, BrainProducts GmbH, Munich, Germany) arranged per the 10–20 system with the impedances kept below 10 kΩ. The EEG was digitized at 500 Hz using BrainAmp amplifiers and BrainVision recorder software (BrainProducts GmbH, Munich, Germany) with an initial reference at FCz. The data was then re-referenced offline using the Reference Electrode Standardization Technique to standardize the reference of scalp EEG recordings to a point at infinity that, being far from all possible neural sources, acts like a neutral virtual reference (Yao, 2001; Dong et al., 2017). To avoid corrupting the raw data, we manually extracted segments of artifact-free EEG data by visually identifying and then removing vertical eye movements, blinks, muscle activity, and other artifact sources. From 40,000 data points, we extracted at least 20,000 artifact-free data points from the resting state task, and from 14,000 data points we extracted at least 12,000 artifact-free data points from the action observation task. For each of the 2-s action observation segments, we then computed the integrated power in the 8–13 Hz range using a Fast Fourier Transform (FFT) performed at 0.2 s intervals with an overlap of 0.25 s (using a Hamming window with 25% overlap). Finally, we averaged the segments over all trials and calculated Mu desynchronization scores with the following formulas: $(ln\left(\mu_{task\;power}\right)\;-\;\ln(\mu_{pre-task\;resting\;power}))/\ln{\left(\mu_{pre-task\;resting\;power}\right)}^{*}100$.

### Multiscale entropy (MSE) analysis

MSE analysis (Costa et al., 2002, 2005) was developed to estimate sample entropy in multiple time scales by using a coarse-graining procedure. MSE analysis uses sample entropy (SE) because it provides greater consistency and is less dependent on a given signal length compared with other entropy methods (Richman and Moorman, 2000). MSE calculation can be summarized in the following three steps: (a) constructing coarse-grained time series per different scale factors (SF); (b) quantifying the SE of each coarse-grained time series; and (c) examining the sample entropy profile over a range of scales. Per this method, the length of each coarse-grained time series is equal to the length of the original time series divided by the SF. For Scale 1, the time series is merely the original time series.

The MSE analysis of EEG signal has been described previously (Catarino et al., 2011) using parameters of *m* = 2, *r* = 0.15, and a SF up to 40. In this study, we used *m* = 2, *r* = 0.15 standard deviation (SD), *N* = 14,000 data points and SF of 20 for resting state, and *N* = 14,000 data points and SF of 20 for the action observation task. We N/*SF* = 14,000/20 = 700 data points, which is enough to obtain a reliable estimation of the SE values (Richman and Moorman, 2000).

Using the MSE of resting-state EEG signal as the reference, we classified MSE profiles into three types: (1) increased complexity (i.e., increased entropy in all scales), (2) reduced complexity toward regularity (i.e., decreased entropy in all scales), or (3) reduced complexity toward randomness (i.e., increased entropy in fine scales followed by decay in entropy as the scale factors increase; Yang et al., 2015). The random type of MSE profile quantifies uncorrelated randomness that cannot be fully captured by single-scale entropy.

### Network analysis

**Figure 2:**
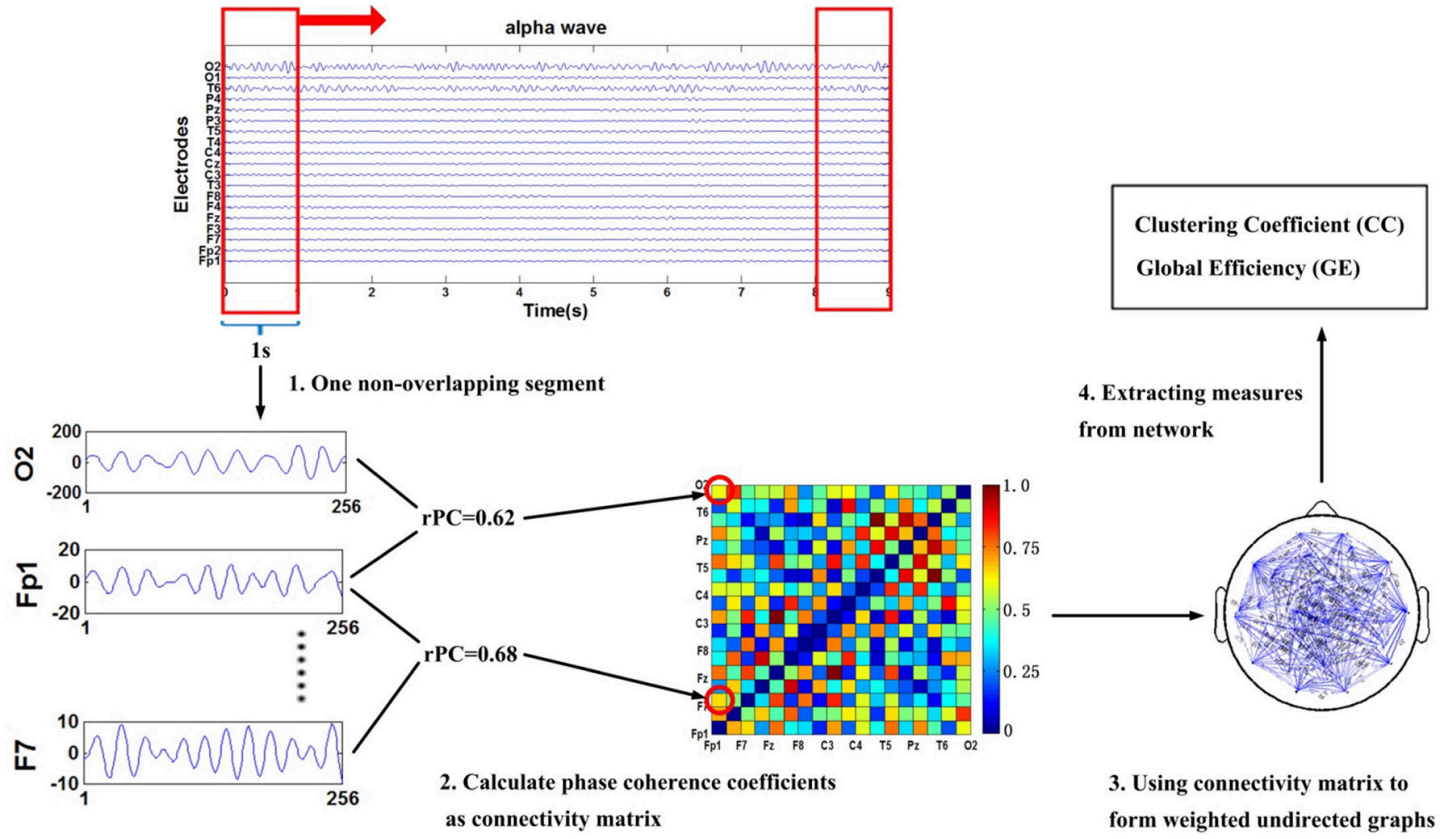
The multi-step illustration of the procedure for extracting networks' measures from EEG time series to sub-network. To be noted, the EEG series given in this graph are the 10th subject's resting state data set and the electrode Fp1, F7, and O2 are shown as an example of the phase synchronization coefficients computed between two electrodes in the alpha band. Additionally, the graph is fully connected. Abbreviation: rPC: respective phase coherence.

We used the first 2 s from the eyes-open baseline and the first of seven segments from the action observation task, for analysis. We then calculated the respective phase coherences (rPCs) for every electrode pair to obtain network measures (please see Figure 2 for a graphical illustration of the procedure). The method used for obtaining the rPC is the phase-locking index (Tass et al., 1998). Once the rPC values between all the electrode-pairs were calculated, we obtained an undirected and weighted network by regarding each electrode as a node and the rPC values as the weight between two corresponding nodes. Then we considered two network measures, clustering coefficient (CC; Rubinov and Sporns, 2010) and global efficiency (GE; Rubinov and Sporns, 2010) to investigate the topological characteristics of the network. Based on graph theory, in the undirected and weighted network with *n* nodes in a nodes' set ***N***, CC of a node j is obtained by the ratio of geometric mean of triangles around the node to the maximum possible number of the connections between all the neighbors of the node, as

(1) $$\begin{array}{r} CC_{j}=\frac{1}{k_{j}\left(k_{j}-1\right)}\overset{N}{\underset{i,m=1}{\sum }}{\left(w_{ij}w_{im}w_{jm}\right)}^{1/3}\;, \end{array}$$

where

(2) $$\begin{array}{r} k_{j}=\;\overset{N}{\underset{i=1}{\sum }}w_{ij} \end{array}$$

is the weighted degree of node j, *w*_*ij*_ are connection weights which are normalized between 0 and 1 for all i and j related to edges (*i, j*), and *N* is the number of all nodes. Importantly, the clustering coefficient can indicate the degree of local interconnectedness of a node and applied to evaluate the local structure of a graph (Rubinov and Sporns, 2010; Miraglia et al., 2017; Wang and Tao, 2017). The average value of all nodes' CC (denoted as aver_CC in this paper) is the average clustering coefficient CC over all nodes, as expressed in Equation (1).

The global efficiency that allows the existence of isolated points is the reciprocal of the shortest path, and not only reflects the global traffic capacity and integration, but also evaluates the performance of the graph effectively (Achard and Bullmore, 2007). The definition of global efficiency (denoted as GE) is given in Equation (3):

(3) $$\begin{array}{r} GE=\frac{1}{N\left(N-1\right)}\;\overset{N}{\underset{i,j,i\neq j}{\sum }}\frac{1}{d_{ij}}, \end{array}$$

The shortest weighted path length between node i and j is defined as Equation (2):

(4) $$\begin{array}{r} d_{ij}=\sum_{a_{uv}\in gi\overset{w}{↔}j}f\left(w_{uv}\right),\; \end{array}$$

where *f* is a map (e.g., an inverse) from weight (*w*_*uv*_) to length, and *g*_*ij*_ is the shortest weighted path between node i and j (Rubinov and Sporns, 2010).

### Statistical analysis

Statistical analyses were carried out using R version 3.3.2 (R Core Team, 2016). We set the alpha significance value at .05 and confirmed the normal distribution of the MSE values using the Kolmogorov–Smirnov normality test and by examining skewness and kurtosis values, for the (C3, C4, CZ, F3, F4, FZ, P3, P4, PZ, O1, O2, and OZ) electrodes. We found all MSE scores to be normally distributed. To determine the complexity score of the mu signal, we calculated the average sample entropy across all scale factors. All analyses were performed on a subset of electrodes reflecting neural activation in the sensorimotor cortex (C3, C4, and CZ as a control), which have been shown to produce the strongest mu signal, and frontal (F3, FZ, and F4), parietal (P3, PZ, and P4), and occipital (O1, OZ, and O2) areas as control regions. Repeated measures ANOVAs were carried out using the “car” package (Fox et al., 2012). Collapsing across the original experimental task conditions, we averaged MSE and mu power across all warmth/competence conditions to collapse the data into a single motor observation condition. We used Bonferroni correction to control for multiple comparisons, for 12 electrode comparisons the corrected *p-value* = *0.05/12* = *0.004*.

## Results

### Mu desynchronization

We confirmed that significant mu desynchronization had occurred in our region of interest using a series of one sample *t*-tests on desynchronization scores obtained from electrodes C3 [*t*_(44)_ = −*5.209, p *<* 0.001, d* = *0.759*] and C4 [*t*_(44)_ = −*3.117, p* = *0.003, d* = *0.465*], and that no significant alpha desynchronization occurred in the occipital electrodes O1 [*t*_(44)_ = −*0.421, p* = *0.676*]*, Oz* [*t*_(44)_ = *0.043, p* = *0.966*], or O2 [*t*_(44)_ = *1.526, p* = *0.134*]. These findings suggest that we indeed picked up mu desynchronization due to MR and not changes in alpha related to attentional shifts. Additionally, we performed a 3-way repeated measures ANOVA with Mu/alpha desynchronization as the dependent variable, and electrode centrality [frontal (F3, F4, Fz), central (C3, C4, Cz), parietal (P3, P4, Pz), and occipital (O1, O2, and Oz) electrodes], and electrode lateralization (left, central, right) as the within-subject factors. We found a significant main effect of lateralization [*F*_(2_, _88)_ = *40.733, p *<* 0.001*], no effect of centrality [*F*_(3_, _132)_ = *1.152, p* = *0.331*], and a significant interaction effect between lateralization and centrality [*F*_(6_, _264)_ = *37.557, p *<* 0.001*]. Unpacking the interaction using simple effects, we found significantly more desynchronization at the C3 electrode compared to posterior P3, [*F*_(1_, _44)_ = *9.133, p* = *0.004*], Pz [*F*_(1_, _44)_ = *32.023, p *<* 0.001*], P4, [*F*_(1_, _44)_ = *40.321, p *<* 0.001*], occipital, O1, [*F*_(1_, _44)_ = *42.602, p *<* 0.001*], Oz, [*F*_(1_, _44)_ = *18.302, p *<* 0.001*], and O2, [*F*_(1_, _44)_ = *28.230, p *<* 0.001*], and frontal, Fz [*F*_(1_, _44)_ = *28.161, p *<* 0.001*], F4 [*F*_(1_, _44)_ = *30.646, p *<* 0.001*], F3 [*F*_(1_, _44)_ = *28.336, p *<* 0.001*] electrodes, further confirming that mu-desynchronization was region specific. An additional one sample *t*-test was performed to check for beta desynchronization and no significant beta desynchronization was found [*t*_(44)_ = –1.101, *p* = 0.277]. In sum, these findings suggest that desynchronization of mu was primarily localized to the C3 electrode–our region of interest.

### Multiscale entropy (MSE) analysis

**Figure 3:**
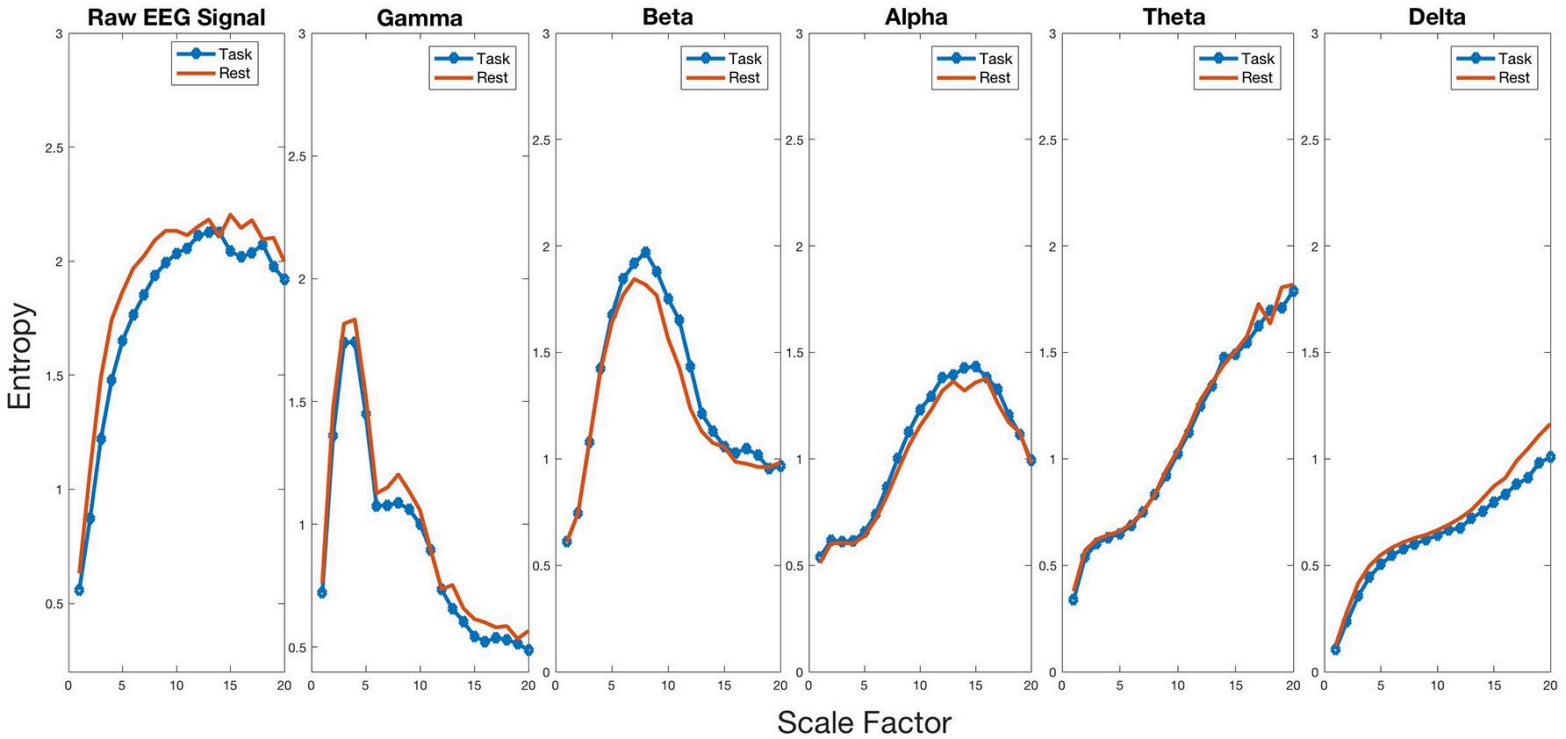
Complexity profiles of the raw signal and all frequency bands for baseline and task.

To test for changes in entropy in the mu frequency band during MR, we performed a paired *t*-test on our electrode of interest (C3). We found average entropy to be higher during the task (*mean* = *1.057, SD* = *0.097*) compared to rest [*mean* = *1.004, SD* = *0.129, t*_(44)_ = *3.524, p* = *0.001, d* = *0.525*], indicating increased complexity of the mu signal. To determine whether entropy change during MR was frequency-band specific, we performed a series of paired *t*-tests on the five frequency bands (gamma = 30–60 Hz, beta = 13–30 Hz, alpha = 8–13 Hz, theta = 4–8 Hz, and delta = 1–4 Hz) between rest and task. After Bonferroni correction, we found significant changes in entropy for all frequency bands, as shown in Figure 3. Gamma *(baseline, m* = *0.936, SD* = *0.085; task, m* = *0.846, SD* = *0.106)* [*t*_(44)_ = *5.892, p *<* 0.001, d* = *0.878*], beta *(baseline, m* = *1.352, SD* = *0.073; task, m* = *1.311, SD* = *0.073)* [*t*_(44)_ = *3.279, p* = *0.002, d* = *0.489*], theta *(baseline, m* = *1.124, SD* = *0.051; task, m* = *1.029, SD* = *0.071)* [*t*_(44)_ = *7.636, p *<* 0.001, d* = *1.138*], and delta *(baseline, m* = *0.704, SD* = *0.041; task, m* = *0.626, SD* = *0.063)* [*t*_(44)_ = *8.307, p *<* 0.001, d* = *1.238*] all showed a decrease in entropy for task compared to rest. These findings indicate that complexity change has a unique increase in the alpha band while all other frequencies showed a significant decrease in complexity.

To determine whether MSE change in the alpha band during MR was region specific, we performed a 3-way repeated-measures ANOVA with average entropy as the dependent variable, and condition (resting vs. task) and electrode (C3, C4, Cz, F3, F4, Fz, P3, P4, Pz, O1, O2, and Oz) as the within-subject factors. We found significant main effects for electrode [*F*_(11_, _484)_ = *10.981, p *<* 0.001*], condition [*F*_(1_, _44)_ = *8.791, p* = *0.005*], and an interaction between condition and electrode [*F*_(11_, _484)_ = *2.003, p* = *0.026*]. To unpack the interaction, we performed pairwise comparisons for each electrode showing significant increases in average entropy in the C3 [*F*_(1_, _44)_ = *12.468, p *< *0.001*, $\eta_{p}^{2}$ = *0.220*] and C4 [*F*_(1_, _44)_ = *17.953, p *<* 0.001*,${\;\eta }_{p}^{2}$ = *0.290*] electrodes. This finding suggests that changes in MSE in the Mu frequency band between baseline and task only occur in the bilateral sensorimotor area, with no changes to occipital regions with alpha activity.

To determine whether entropy change in the alpha band during MR was dependent on the SF, we performed a 3-way repeated-measures ANOVA with entropy as the dependent variable, and condition (resting vs. task) and SF (1 through 20) as the within-subject factors. A significant condition by SF interaction effect was found [*F*_(19_, _798)_ = *15.397, p *<* 0.001*]. This finding indicates that the sample entropy curves of each condition presented a different slope as the scale factor increased. Although the difference between conditions is not noticeable for smaller scale factors, the curves for both conditions become distinguishable for higher scale factors, representing greater differences in sample entropy at higher scale factors.

To determine whether complexity of the mu signal predicts MR, we used multiple linear regression models to examine the relationship between MR and baseline entropy. Using general linear hypothesis testing on mu desynchronization scores, the best model fit was found for model 1 with a significant regression equation [*F*_(1_, _43)_ = *11.73, p* = *0.001, with an R*^2^ *of 0.214*], as shown in Table 1. The model indicates that resting state entropy is significantly predictive of MR such that higher baseline entropy predicts less mu desynchronization, indicating less MR.

**Table 1:** Parameter estimates, approximate *p*-values, and associated goodness-of-fit statistics for a series of models depicting the relationship between mu desynchronization scores (as an index of motor resonance) and complexity of the mu rhythm.

**Predictor**	**Model 1 ß(SE)**	**Model 2 ß(SE)**	**Model 3 ß(SE)**
Intercept	−77.88^***^ (19.29)	−41.90 (29.16)	−4.929 (7.597)
C3 resting complexity	65.17^**^ (19.03)		
C3 task complexity		27.95 (27.43)	
Baseline Mu power			−3.839 (3.708)
**MODEL FIT STATISTICS**			
*R*^2^	0.214	0.024	0.024
RMSE	15.98	17.82	17.81
DF	43	43	43

*** *p < 0.001*,

** *p < 0.01. RMSE, root mean square error; SE, standard error of the mean; DF, degrees of freedom*.

### Efficiency analysis

**Figure 4:**
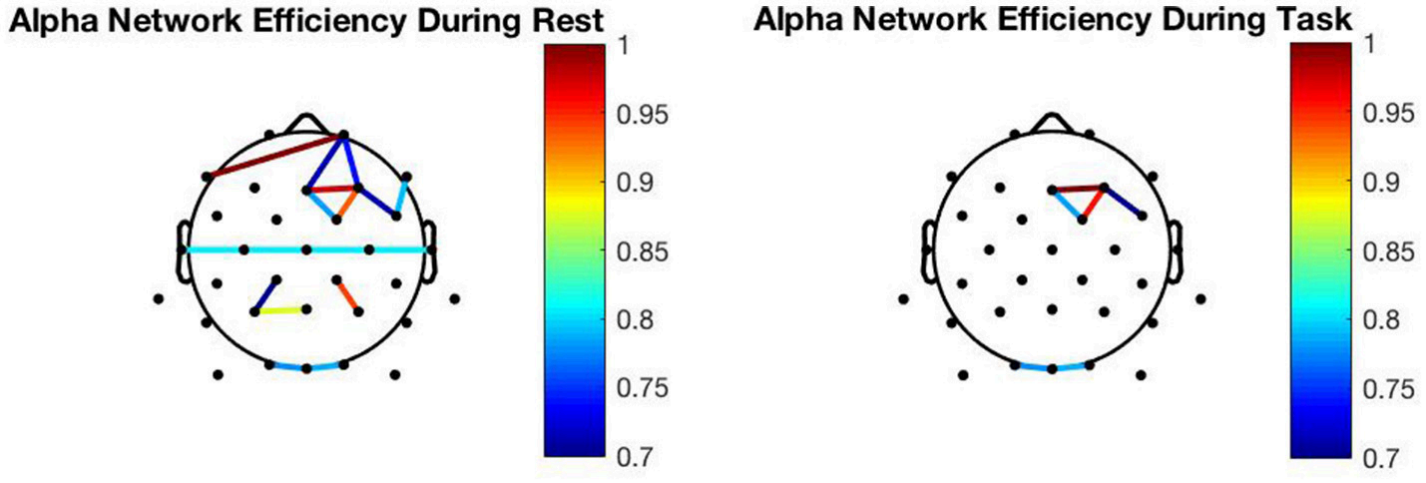
Network changes: Resting alpha network connectivity (Left) Alpha network connectivity during action observation (Right). To be noted, the graphs presented are from the 10th subject.

To explore the relationship between network efficiency and MR, we performed a 3-way repeated measures ANOVA with global efficiency as the dependent variable, and condition (resting vs. observation), and frequency band (gamma, beta, alpha, theta, delta) as the within-subject factors. We found a significant main effect for frequency [*F*_(4_, _176)_ = *64.452, p *<* 0.001*, $\eta_{p}^{2}$ = *0.593*] and condition [*F*_(1_, _44)_ = *5.723, p* = *0.021*, $\eta_{p}^{2}$ = *0.113*] and no significant interaction effect for condition and frequency [*F*_(4_, _176)_ = *0.934, p* = *0.446*]. We decided to perform pairwise comparisons for each frequency band to determine whether global efficiency modification was frequency specific during MR. The analysis revealed a significant decrease in efficiency in the alpha [*F*_(1_, _44)_ = *15.509, p *<* 0.001*, $\eta_{p}^{2}$ = *0.260*] frequency band only. These findings indicate that global efficiency decreases during action observation in the alpha network only. As shown in Figure 4, alpha connectivity was reduced in frontal, central and parietal areas.

To explore the relationship between the cluster coefficient over the sensorimotor area and MR, we performed pairwise comparisons for the alpha frequency band. A significant decrease in the CC was shown in the alpha band [*t*_(44)_ = *2.879, p* = *0.006, d* = *0.439*] during the task condition. These findings indicate that the local efficiency over the C3 electrode decreases during action observation in the mu frequency band.

Finally, to determine whether there is a relationship between complexity change, measured as the percent change in entropy from baseline to task, and percent change in task efficiency in the alpha band, we performed a simple correlation, finding that there is a significant negative relationship between entropy and global efficiency during the task [*t*_(43)_ = −*2.653, p* = *0.011, r* = −*0.375*] suggesting that there may be a direct relationship between efficiency change and complexity change.

## Discussion

The change in amplitude in the mu frequency band over the sensorimotor area, resulting from the observation of object-oriented actions, has been used as a marker of MR (Fox et al., 2015). The intention of this study was to explore how nonlinear brain dynamics change during MR, and how these changes are related to the linear changes observed in the mu power spectrum. We found that MSE measured from the C3 electrode over the sensorimotor area during action observation, increases in the mu frequency band only. At the same time, the MSE of the raw signal and other frequency bands decreases in response to action observation. These findings confirm our hypothesis that a decrease in the mu power spectrum results in increased complexity in that same frequency band, and the increase is frequency specific. We did not predict decreased complexity in the other frequency bands and this phenomenon should be addressed in future studies. The respective decrease in global efficiency in the alpha band during MR, may indicate greater local information processing due to a release from so-called alpha inhibition. The increased complexity in the mu band and decreased global alpha efficiency may underlie an increase to local functional integration (Sporns et al., 2000). Further supporting the link between increased complexity and MR, we found that larger decreases in mu power are associated with greater increases to the MSE of that signal.

Oscillations within specific frequency bands h ave been considered to be associated with information processing (Başar et al., 2001; Ghanbari et al., 2015), and our findings suggest that information processing increases in the mu band during MR. Interestingly, Ghanbari et al. (2015) found a significantly negative relationship between change in alpha connectivity and change in MSE in patients with ASD, but no such relationship in the healthy controls. We did not find any significant relationship between complexity change and network efficiency change, confirming this finding for healthy controls. It is important to note, that the decrease in entropy within the other frequency bands does not necessarily indicate a reduction in information processing (McDonough and Nashiro, 2014), but instead may reflect that the flow of information in the sensorimotor area being mainly regulated by the alpha band during action observation.

Interestingly, the differences in the alpha complexity profiles of rest and task condition appear to be mainly in the higher time scales. Greater entropy at high scale factors are believed to capture long-range temporal correlations (Bhattacharya et al., 2005). Bhattacharya et al. (2005) found that neurons that showed long-range correlations also showed statistically significantly correlated firing, suggesting that the presence of long-range correlations indicates a memory of the firing pattern. The decrease in long-range correlations in the mu band reflects we found, may therefore reflect greater non-random variability in the underlying neuronal firing patterns, and therefore greater complexity.

### Can EEG complexity predict motor resonance?

We expected MSE measured in the mu band over the sensorimotor area to increase during action observation due to less synchronous firing, reflecting an increase of the non-random variability within the underlying patterns of activity. Our analyses confirmed this hypothesis showing that a greater percent increase in MSE from baseline to action observation in the mu band was significantly associated with a greater decrease in the mu power spectrum, indicating greater MR. We also hypothesized that resting MSE measured in the mu band would predict decreases in the mu power spectrum during action observation, reflecting adaptability. Complexity may be a reflection of the adaptability to a constantly changing environment (Hager et al., 2016), and it may be that the level of MSE at rest predicts the adaptability to information processing when viewing an action. Specifically, we found that higher levels of resting MSE in the mu band were predictive of less mu desynchronization during action observation. This finding suggests that measuring signal complexity in the mu band at rest may have the potential to serve as a predictor of adaptability to a stimulus intended to trigger mu desynchronization.

### EEG network changes

The observed increase in MSE during action observation was related to a decrease in global efficiency of the alpha network. If the role of the alpha rhythm is indeed inhibitory (Klimesch et al., 2007; Jensen and Mazaheri, 2010; Klimesch, 2012), this finding may suggest that signal complexity measured in the mu band in the sensorimotor area acts as a mediator of release from alpha inhibition. We found decreased network efficiency in the alpha band during MR, and no change in the gamma, theta, and delta bands. The reduced global efficiency in the alpha band suggests a reduction in the exchange of information across the brain in that band. Klimesch (2012), posited that the magnitude of alpha desynchronization reflects the degree of cortical activation because lower alpha power releases inhibition, and we found a stronger relationship between MSE and mu desynchronization when global alpha efficiency was lower during action observation. Since alpha oscillations are associated with inhibiting neural networks (Klimesch et al., 2007; Jensen et al., 2014), our finding of increased complexity in the mu band during mu desynchronization and decreased global alpha network efficiency, may indicate decreased alpha inhibition during action observation; an unattainable observation when looking at the power spectrum alone.

## Conclusions

Our current study suggests that the desynchronization of mu over the sensorimotor area during the observation of an object-oriented action results in previously unexplored changes to nonlinear brain dynamics. The degree to which MSE measured in the mu band increases during action observation, may be related to individual differences in basal complexity leading to a dampening of local alpha and global alpha inhibition. Our findings suggest that increased complexity in the sensorimotor area reflects an increase of local information capacity, thus enabling successful processing of stimulus-related information, by triggering a decrease in alpha inhibition. These findings encourage future incorporation of measures of nonlinearity into analysis of MR to improve understanding of how the brain processes information.

## Author contributions

BH and JG conceived of the presented idea. BH developed the theory and performed the computations. AY and JG verified the analytical methods. JG supervised the findings of this work. BH spearheaded writing the manuscript. All authors discussed the results and contributed to the final manuscript.

### Conflict of interest statement

The authors declare that the research was conducted in the absence of any commercial or financial relationships that could be construed as a potential conflict of interest. The reviewer MI and handling Editor declared their shared affiliation.
